# Evaluating the Prebiotic Properties of Agar Oligosaccharides Obtained from the Red Alga *Gracilaria fisheri* via Enzymatic Hydrolysis

**DOI:** 10.3390/plants12233958

**Published:** 2023-11-24

**Authors:** Jantana Praiboon, Sudathip Chantorn, Weerada Krangkratok, Pradtana Choosuwan, Orawan La-ongkham

**Affiliations:** 1 Department of Fishery Biology, Faculty of Fisheries, Kasetsart University, Chatuchak, Bangkok 10900, Thailand; 2 Department of Biotechnology, Faculty of Science and Technology, Thammasat University, Rangsit Centre, Pathum Thani 121200, Thailand; 3 Institute of Food Research and Product Development, Kasetsart University, Bangkok 10900, Thailand

**Keywords:** agarophyte, functional ingredient, lactic acid bacteria, pathogen inhibition, gastrointestinal condition

## Abstract

Currently, the demand in the food market for oligosaccharides with biological activities is rapidly increasing. In this study, agar polysaccharides from *Gracilaria fisheri* were treated with β-agarases and hydrolyzed to agar oligosaccharides (AOSs). High-performance anion-exchange chromatography/pulsed amperometric detection (HPAEC-PAD), Fourier-transform infrared spectroscopy (FT-IR), and gel permeation chromatography (GPC), were employed to analyze the chemical characteristics of AOSs. The FT-IR spectra revealed that the enzymatic hydrolysis had no effect on specific functional groups in the AOS molecule. To investigate the prebiotic and pathogen inhibitory effects of AOSs, the influence of AOSs on the growth of three probiotic and two pathogenic bacteria was examined. The gastrointestinal tolerance of probiotics in the presence of AOSs was also investigated. AOSs enhanced the growth of *Lactobacillus plantarum* by 254%, and inhibited the growth of *Bacillus cereus* by 32.80%, and *Escherichia coli* by 58.94%. The highest survival rates of *L. plantarum* and *L. acidophilus* were maintained by AOSs in the presence of α-amylase and HCl under simulated gastrointestinal conditions. This study demonstrates that AOSs from *G. fisheri* exhibit potential as a prebiotic additive in foods.

## 1. Introduction

Currently, people are focusing more on their health. In addition to exercising, consumers are increasingly focused on choosing good and functional foods. Food must be consumed for nutrition to prevent or reduce the risk of disease and to strengthen the body’s immune system. Prebiotics are foods that cannot be absorbed in the digestive system of the human body but are digested by bacteria in the colon. These foods stimulate the functions and promote the growth of probiotic microorganisms (probiotics), which have a positive effect on health. Prebiotics are found in many vegetables, fruits, and algae, such as onions, bananas, cereals, beans, garlic, artichokes, chicory, asparagus, mushrooms, brown seaweed, red seaweed, and green seaweed [1].

Seaweeds are known to be rich in various types of polysaccharides, such as agar, alginate, carrageenan, fucoidan, and ulvan, which generally exhibit soluble fiber and prebiotic properties [2]. These compounds cannot be digested via the enzymes of the human upper gastrointestinal tract and promote beneficial bacterial growth by serving as substrates for fermentation in the colon, resulting in the production of short-chain fatty acids, which have multiple functions and contribute to maintaining health. They also modulate intestinal metabolism, including fermentation, inhibit pathogens, and treat inflammatory bowel disease [3,4]. However, seaweed polysaccharides generally have a high molecular weight, which leads to poor solubility and low bioavailability. Therefore, the depolymerization process to produce oligosaccharides is necessary to improve the deficiencies of water insolubility and bioavailability [2,4,5].

Various depolymerization processes have been used to reduce the size of polysaccharides, including chemical depolymerization (acid hydrolysis and H_2_O_2_-induced depolymerization) [6,7,8], and enzymatic hydrolysis [9,10,11,12]) and physical depolymerization (e.g., thermal, microwave, γ-irradiation, and ultrasonication) [13,14]. Chemical depolymerization can produce undesirable by-products, such as furfural, hydroxymethylfurfural (5-HMF), and large amounts of reducing sugars, when polysaccharides are hydrolyzed with strong acids at high temperatures over several hours. When added to food, these by-products can affect the color, odor, and taste of foods; thus, applications of oligosaccharides obtained via chemical processes in functional food applications are limited. The hydrolysis of polysaccharides with specific enzymes that hydrolyze the glycosidic bonds, on the other hand, leads to stereospecific oligosaccharides. Therefore, depolymerization of polysaccharides using enzymes is the best method to produce oligosaccharides for use in functional foods [4]. Ulvan lyase (PL24, PL25, and PL28), alginate lyase, fucoidanase, glucanase, laminarinase, β-Mannanase, β-galactosidase, α-agarase, β-agarase, β-porphyranase, β-galactosidase, κ-carrageenase, ι-carrageenβase, λ-carrageenase, cellulase, and pectinase are specific hydrolytic enzymes that have also been successfully employed to produce oligosaccharides from seaweed. These enzymes can be produced by microorganisms from many sources, including seawater, seaweed, shellfish, soil, and the human gut [5,6,10,11,15,16,17,18,19].

*Gracilaria* spp. is a red alga found worldwide and is commonly used as the main raw material (80%) in the agar industry, followed by *Gelidium* spp. (20%) [20,21]. *Gracilaria fisheri* is widespread in Southern Thailand and has previously been described as abundant. Currently, *G. fisheri* is abundantly cultivated and popular as shrimp pounds. The annual productivity of *G. fisheri* is approximately 100–200 tons of dry weight per year, with a retail price of USD 1 per 0.3–0.5 kg wet weight [22]. Agar is a hydrocolloid, which can form a gel in water. Agar is a mixture of two polysaccharides, namely agarose and agaropectin. The agarose molecule consists of β-D-galactopyranose and 3,6-anhydro-α-L-galactopyranose residues linked via alternating 1→3 and 1→4 glycosidic bonds. Agaropectin is structurally similar to agarose, except that α-L-galactose 6-sulfate is sometimes present instead of 3,6-anhydrogalactose, and hydroxyl groups are replaced by a sulfate, a methoxyl, or a pyruvic acid group [23]. In addition, agar oligosaccharides (AOSs) obtained from the degradation of agar polysaccharides (APSs) have been reported for various biological activities, such as antioxidant, anticancer, anti-inflammatory, anti-melanogenesis, immunomodulatory, antilipidemic, and hypocholesterol activities, and exhibit prebiotic properties [7,9,10]. However, a few studies have reported that AOSs can stimulate the growth of beneficial microorganisms in the body and help strengthen the immune system [10,15,24,25]. To the best of our knowledge, the prebiotic potential of AOSs from *G. fisheri* has not been well studied. Understanding the prebiotic properties and physicochemical properties of AOSs is crucial for their successful application in functional foods. In this study, we produced AOSs from *G. fisheri* via enzymatic hydrolysis and investigated the prebiotic properties of AOSs. In addition, the physicochemical properties of AOSs were investigated to provide information for the possible use of AOSs as an ingredient in food products.

## 2. Results

### 2.1. Yield Chemical Composition of APSs

The yield of APSs was 39.10 ± 0.23% of the dry weight of algae. The chemical composition of APSs consisted of 44.94 ± 1.89% carbohydrate, 3.76 ± 0.32% sulfate, and 21.54 ± 1.19% uronic acid. In addition, a small amount of protein (0.81 ± 0.02%) was also found in APSs.

### 2.2. Agar Oligosaccharide (AOS) Production

#### 2.2.1. Sugar and Oligosaccharide Contents

**Figure 1:**
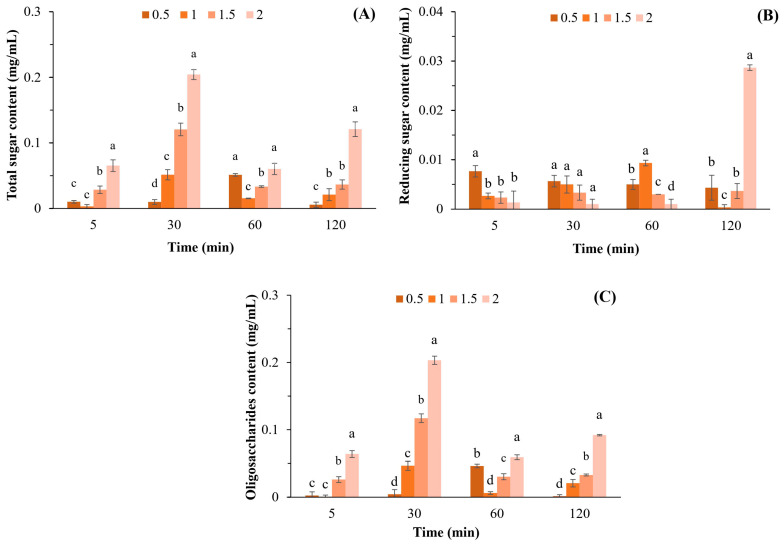
Total sugar (**A**), reducing sugar (**B**), and oligosaccharide (**C**) contents after hydrolyzing APSs at various concentrations (0.5–2.0% *w*/*v*) by β-agarase. Data are presented as the mean of triplicate determination ± standard deviation. Different letters above the bars indicate a significant difference (*p* ≤ 0.05).

The highest total sugar content (0.204 mg/mL) was achieved after incubation of 1 mg/mL APSs with β-agarase for 30 min (Figure 1A), which was significantly different compared to other APS concentrations. Based on these results, the total sugar content was determined using the APS concentration and not by the reaction time, although the interaction between APSs and β-agarase was increased.

The highest level of reducing sugars (0.029 mg/mL) was obtained via incubation of 2 mg/mL APSs with β-agarase for 120 min (Figure 1B). Moreover, the highest oligosaccharide content of 0.203 mg/mL was obtained through incubating 2 mg/mL APSs with β-agarase for 30 min, with a statistical difference compared to the oligosaccharide content at other APS concentrations observed (Figure 1C).

#### 2.2.2. The Monosaccharide Composition of APSs and AOSs

Based on HPAEC-PAD analysis of the monosaccharide constituents in the APSs of *G. fisheri*, galactose was the major constituent (74.92%), followed by minor amounts of glucose (12.58%), rhamnose, mannose, and xylose as other constituents (Table 1). None of the APSs contained arabinose in their compositions.

#### 2.2.3. Molecular Weights of APSs and AOSs

APSs and AOSs obtained from *G. fisheri* were analyzed for their molecular weights via GPC and compared with standard pullulan (MW 180–805,000 Da). The results showed that APSs had an average molecular weight, with a Mn of 1.355 × 10^4^ Da and a Mw of 4.816 × 10^6^ Da, and when APSs were hydrolyzed with β-agarase, AOS mixtures with smaller molecular sizes were obtained compared with APSs. In addition, it was found that AOSs in the mixture consisted of two peaks, with AOSs being the most abundant molecular size (50.62%) with an average molecular weight (Mn) of 1346 Da and a Mw of 1756 Da, followed by 49.38% of AOSs with a Mn of 2.215 × 10^4^ Da and a Mw of 2.715 × 10^4^ Da (Table 2).

#### 2.2.4. FT-IR Spectra of APSs and AOSs

**Figure 2:**
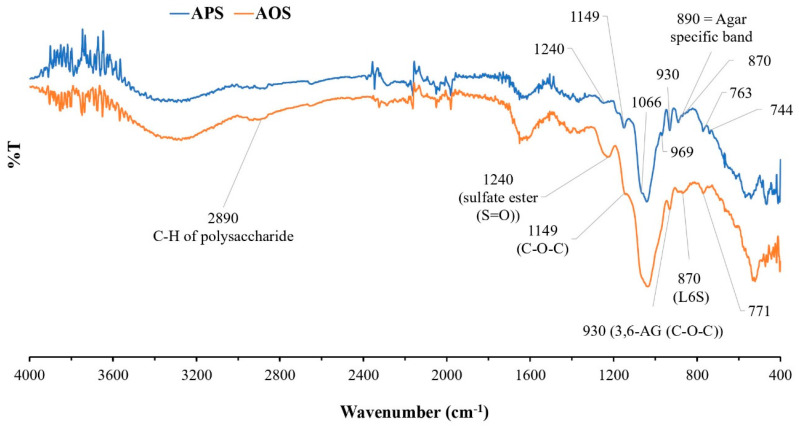
FT-IR spectra of agar polysaccharides (APSs) and agar oligosaccharide (AOSs).

The chemical structure and functional groups of APSs and AOSs were analyzed via FT-IR spectroscopy. The FT-IR spectra of APSs and AOSs exhibited the same characteristics. The strong signals at 1240 cm^−1^, 1066 cm^−1^, 930 cm^−1^, and 890 cm^−1^ corresponded to typical features of agar (Figure 2). Signals in the range of 800–900 cm^−1^ corresponded to the sulfated group substitution in the structure. The signal at 930 cm^−1^ represented the C-O-C bridge of the 3,6-anhydro-α-L-galactose group. The signal at 1240 cm^−1^ contributed to the sulfate group (S=O) in a molecule. The signals at 763 cm^−1^, 771 cm^−1^, 890 cm^−1^, and 1066 cm^−1^ were assigned to agar-specific polysaccharide signals. The signal at 870 cm^−1^ represented a sulfate substitution in the C-6 position of L-galactose (L-galactose-6-sulfate (L6S)) in the agar structure. The signal at 2890 cm^−1^ contributed to the C-H stretching vibration of polysaccharides in general [26,27,28,29,30].

### 2.3. AOSs’ Prebiotic Properties

#### 2.3.1. Effect of AOSs on LAB Growth

**Figure 3:**
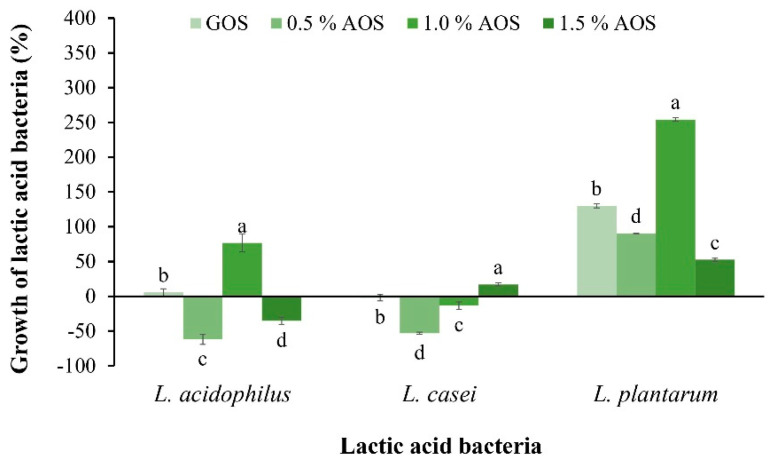
Effect of AOSs (0.5, 1.0 and 1.5%) and GOSs on the growth of *L. acidophilus*, *L. casei*, and *L. plantarum*. Data are presented as the mean of triplicate determination ± standard deviation. Different letters above the bars indicate a significant difference (*p* ≤ 0.05).

The effect of AOSs on the growth of *Lactobacillus acidophilus*, *L. casei*, and *L. plantarum*, was investigated by comparing the percentage increase in LAB with the control (without sample) and GOSs, a commercial prebiotic. The results revealed that 1% of AOSs promoted the growth of *L. plantarum* up to 254.21%, which was significantly different from other concentrations and GOSs (Figure 3). However, the effect of AOSs on the percentage increase in *L. acidophilus* and *L. casei* was equally low to that of GOSs, indicating that both LABs could utilize AOSs for growth. AOSs at a concentration of 1.5% promoted the growth of *L. casei* by 17.6%, while AOSs at a concentration of 1.0% promoted the growth of *L. acidophilus* by 76.69%.

#### 2.3.2. Effect of AOSs on the Pathogenic Bacteria

**Figure 4:**
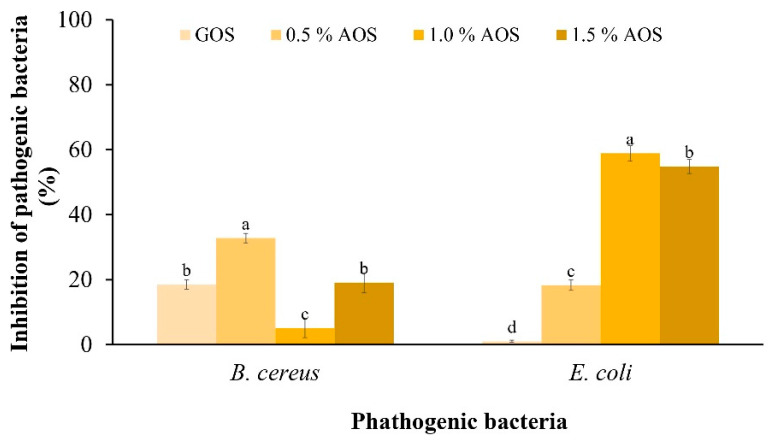
Inhibitory effect of AOSs (0.5, 1.0 and 1.5%) and GOSs on *B. cereus* and *E. coli*. Data are presented as the mean of triplicate determination ± standard deviation. Different letters above the bars indicate a significant difference (*p* ≤ 0.05).

The inhibitory effect of AOSs on the pathogen (Figure 4) showed that AOSs inhibited the growth of both pathogens better than GOSs. In addition, 0.5% of AOSs inhibited the growth of *B. cereus* by 32.80%, which was significantly different from other concentrations. Meanwhile, 1.0% of AOSs inhibited the growth of *E. coli* by 58.94%, which was significantly different from other concentrations and statistically significant under other concentrations.

#### 2.3.3. Protective Effect of AOSs on Probiotics under Simulated Gastrointestinal Conditions

**Figure 5:**
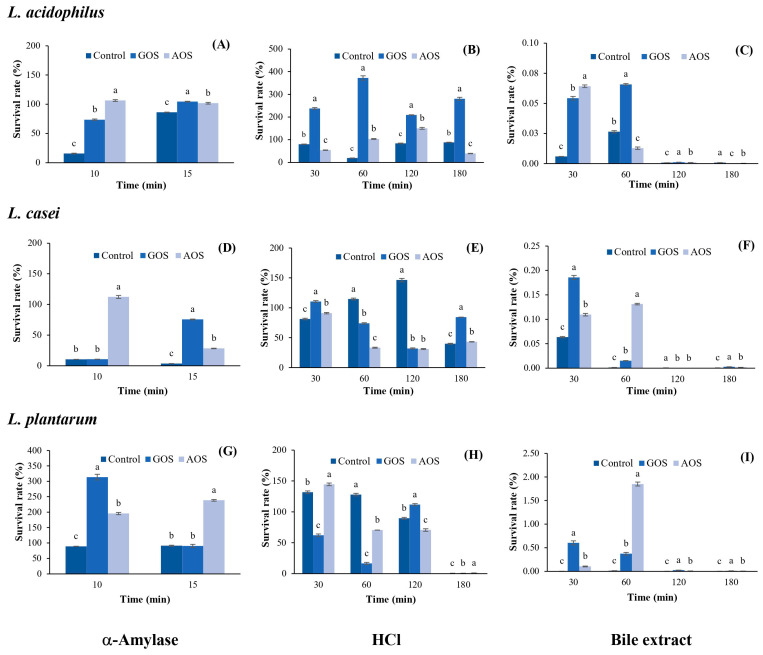
The percentage of survival of *L. acidophilus*, *L. casei*, and *L. plantarum* under the different simulated gastrointestinal conditions. Under α-Amylase conditions (**A**,**D**,**G**); HCl conditions (**B**,**E**,**H**), and bile extract conditions (**C**,**F**,**I**). Data are presented as the mean of triplicate determination ± standard deviation. Different letters above the bars indicate a significant difference (*p* ≤ 0.05). GOS: galacto-oligosaccharides; and AOS; agar oligosaccharide.

The results for the protective effect of AOSs on LAB under simulated gastrointestinal conditions are shown in Figure 5. It was found that AOSs exhibit a greater protective effect on *L. acidophilus* in the presence of α-amylase than that of GOSs and the control after 10 min of incubation, with a survival rate of 106.52%; this rate decreased to 101.83% after 15 min of incubation, which was similar to that of GOSs (104.52%) but higher than the control (86.18%) (Figure 5A). Under HCl conditions, AOSs exhibited a lower protective effect on *L. acidophilus* than that of GOSs during 30–180 min of incubation (Figure 5B). However, AOSs showed the greatest protective effect with a survival rate of 150.33% after 120 min of incubation, which was 1.8 times higher than that of the control (83.56%). In addition, the survival rate of *L. acidophilus* under bile salt conditions was very low (0.01–0.06%) and completely lost after only 120 min of incubation (Figure 5C).

As shown in Figure 5D, AOSs exhibited the greatest protective effect on *L. casei* under α-amylase conditions, maintaining a survival rate of 112.46% after 10 min of incubation, with a significant difference from GOSs (*p* ≤ 0.05). However, the survival rate of *L. casei* decreased to 28.32% after 15 min of incubation. Under HCl conditions, AOSs maintained a 91.13% survival of *L. casei* after 30 min of incubation and remained stable within 33.34–43.21% until 180 min (Figure 5E). In addition, the survival rate of *L. casei* under bile salt conditions was very low (0.11–0.13%) and completely lost after only 120 min of incubation (Figure 5F). AOSs showed a greater protective effect on *L. plantarum* than GOSs in the presence of α-amylase with a survival rate of 238.65% after 15 min of incubation, which was 2.6 times higher than that of GOSs and the control (Figure 5G). Under HCl conditions, the survival rate of *L. plantarum* with AOSs after 30 min of incubation was 144.45%, which was 2.3 times and 1.5 times higher than that of GOSs and the control, respectively. Thereafter, survival remained at approximately 70% for 60–120 min and was completely lost after 180 min of incubation (Figure 5H). Similar to the results for *L. acidophilus* and *L. casei*, the survival rate of *L. plantarum* under bile salt conditions was very low (0.10–1.85%) and completely lost after only 120 min of incubation (Figure 5I).

## 3. Discussion

### 3.1. Chemical Composition and Yield of APSs

As previously established, the polysaccharides extracted from the genus *Gracilaria* are agar polysaccharides containing galactose as their main component. In this study, the yield of polysaccharides from *G. fisheri* (39.10%) was similar to the polysaccharide yield extracted from *G. corticata* and *G. edulis* (38–49%) [31]. The sulfate content of *G. fisheri* (3.76%) in this study was similar to the native polysaccharide extract of *G. fisheri* (4.56%) obtained by Praiboon et al. [27] and similar to that of *G. tenuistipitata* (4.93–5.08%) [32]; however, the content was lower than the sulfate content in the polysaccharide *G. fisheri* (12.7%) obtained by Wongprasert et al. [28]. These results indicate that the species, location, environmental factors, and extraction method have an influence on the yield and chemical constituents of the agar polysaccharides [8,27].

### 3.2. Agar Oligosaccharide (AOS) Production and Characterization

The results obtained after APSs were incubated at a concentration of 0.5–2.0% for 120 min showed that the highest total sugar content was obtained when 1 mg/mL was hydrolyzed with β-agarase for 30 min (Figure 1B). The results also showed that the APS concentration had a greater influence on the total sugar content than the reaction time. We conducted an experiment in which we increased the reaction time between APSs and β- agarase to 300 min. However, the results revealed that the total sugar content remained constant for 120 min and did not increase thereafter. The reducing sugar content was at its highest after 2 mg/mL APSs were hydrolyzed with β-agarase for 120 min (Figure 1B). Substrate concentration and incubation time may be the most important factors affecting enzymatic activity. Therefore, the highest reducing sugar content was obtained by digesting APSs with the highest concentration and the longest incubation time. In addition, the highest oligosaccharide content of 0.203 mg/mL was obtained by incubating 2 mg/mL APSs with β-agarase for 30 min, which corresponded to a yield of 10.15% (*w*/*w*). Jiang et al. [11] reported that the yield of agar oligosaccharides obtained via β-agarases ranges from 3.20% to 93.2%, which varies depending on the concentration and purity of the substrates, specificity of the enzyme, and the conditions employed for incubation.

In this study, GPC was used to investigate the molecular sizes of APSs and AOSs. The results showed that when APSs were hydrolyzed with β-agarase, the mixture of AOSs was smaller than that of the APSs by half (50.62%), as AOSs with Mn were 1345 Da and Mw were 1756 Da, followed by 49.38% of AOSs with Mn and Mw were 2.215 × 10^4^ Da and 2.715 × 10^4^ Da, respectively. Siringoringo et al. [33] reported that the molecular weight of AOSs (DP1–DP9) from *G. fisheri* was in the range of 540–1800 Da. In further studies, the activity of several enzymes could be investigated by studying the hydrolytic efficiency of β-agarase together with α-agarase, since these two enzymes exhibit different specificities to glycosidic linkages.

Based on the investigation of the functional groups in APSs from *G. fisheri* algae using FT-IR and in comparison with previous studies of agar extracts from algae of this genus [26,27,28,29,30], APSs exhibit typical spectra of agar polysaccharides. The signal at 930 cm^−1^ was attributed to 3,6-anhydro-α-L-galactose, and the signals at 766 cm^−1^, 890 cm^−1^, and 1068 cm^−1^ denote the characteristic signals of the agar group polysaccharides (Figure 2). The band at 867 cm^−1^ represented a sulfate group substitution in the C-6 position of L-galactose (L-galactose-6-sulfate (L6S)) in agar structures. The results of this study are consistent with the studies by Praiboon et al. [26] and Wongprasert et al. [28], who also investigated the functional groups of polysaccharide extracts from *G. fisheri*. However, in this study, the bands at 850 cm^−1^ and 825 cm^−1^ represented a sulfate group substitution in the C-4 position of D-galactose (D-galactose-4-sulfate (G4S)) and in the C-6 position of D-galactose-6-sulfate (G6S). This difference may have resulted from the different harvest times used for the raw materials or the different times or different extraction procedures.

The FT-IR spectra of APSs and AOSs did not exhibit different spectral characteristics. The results of this study showed that utilizing β-agarase for agar hydrolysis does not cause changes in the functional groups, especially the sulfate group of the agar, due to the specific cleavage properties of the enzyme. Agarases are a group of enzymes in the glycoside hydrolase (GH) family that catalyze the hydrolysis of agar. This enzyme can be divided into α-agarases and β-agarases according to its cleavage mechanism [6]. α-agarases are specific enzymes that cleave the α-1,3 linkage of agar and produce agar oligosaccharides with 3,6 anhydro-galactose at the reducing end. β-agarases hydrolyze the β-1,4 linkage, causing the formation of neoagaro-oligosaccharides (NAOSs) with β-D-galactose at the reducing end [34].

### 3.3. AOSs’ Prebiotic Properties

#### 3.3.1. Effect on LAB Growth

In the study of prebiotic properties, it was found that AOSs can best promote the growth of *L. plantarum* TISTR 1465, in which 1% AOSs promotes the growth of bacteria the most, with a value of 254.21%. This result indicates that AOSs are well suited as a carbon source for bacterial growth. This result is in accordance with observations by Hu et al. [23], who reported that 1% NAOSs stimulated the growth of *Lactobacilus delbrueckii* subp. *bulgaricus* by 77.6% compared with the control. However, this result is different from Zhang et al. [10], who reported that NAOSs obtained from *Gracilaria* via β-agarase hydrolysis showed a slight effect on promoting the growth of *L. delbrueckii* subsp. *bulgaricus* and *Streptococcus thermophilus*.

#### 3.3.2. Inhibitory Effect on Pathogenic Bacteria

In addition, compared to GOSs, AOSs were more effective in inhibiting pathogens. At 1.0%, AOSs exhibited the highest growth inhibition of *E. coli* ATCC 25,922 at 58.94%. This result is consistent with a report of Hayisama-Ae et al. [25], who investigated synbiotic drinks made from fermented red seaweed (*Gracilaria fisheri*) in combination with *Lactobacillus plantarum* DW12 and found that this combination can inhibit the growth of putrefactive pathogens. K-da et al. [35] prepared AOSs from *G. fisheri* via acid hydrolysis and determined their prebiotic properties in colitis mice. The AOSs promoted short-chain fatty acid (SCFA) production and reduced harmful bacteria (such as *Escherichia coli*), attenuating colitis symptoms and gastrointestinal dysmotility in mice.

#### 3.3.3. Protective Effect on Probiotics under Simulated Gastrointestinal Conditions

The upper digestive tract, beginning with the mouth, contains the enzyme amylase. The stomach contains HCl, and the small intestine contains bile salts. Prebiotic substances must be resistant to degradation or destruction by enzymes and acids in the upper gastrointestinal tract. Therefore, an in vitro experiment was designed to test the prebiotic properties by mixing AOSs with LAB strains and determining their resistance to degradation in the upper gastrointestinal tract by observing the survival rate of LAB.

The results of this study indicated that AOSs maintained the survival rates of *L. plantarum* and *L. acidophilus*, ranging from 101.83% to 235.65%, while *L. casei* had survival rates ranging from 28.32% to 112.46% under α-amylase conditions. When AOSs were incubated together with LAB under HCl conditions for 180 min, the survival rates of *L. acidophilus* and *L. casei* were within 39.04–150.33%, while the survival rate of *L. plantarum* was 70.58–144.54% for 30–120 min and then completely lost after 180 min of incubation. These results are consistent with the report of Zhang et al. [10], who determined the protective effect of neoagro-oligosaccharides (NAOSs) under simulated gastric juice conditions and found that NAOSs maintained the survival rate of *Lactobacillus delbrueckii* subsp. *bulgaricus* at 42.9% after 90 min of incubation; then, the rate decreased to 1.9% after 180 min of incubation.

Under bile extract conditions, all LAB strains growing in the AOS-supplemented medium showed the lowest survival rates. It is known that the bile extract represents the small intestine conditions, where the duodenum contains the bile salts secreted by the pancreas. This process contributes to the digestion of nutrients, as there are glands here that produce gastric juice, and most digestion takes place in this area [36]. Therefore, the activity of the bile extract can reduce the growth of LAB. A good probiotic microorganism should have a bile salt hydrolase that can digest bile salts to survive in the digestive system. In general, bile salt hydrolase is often found in microorganisms of the *Lactobacillus* and *Enterococcus* species [37].

The upper digestive tract begins when food enters the body through the mouth. Food then passes through the esophagus into the stomach and duodenum. Prebiotic substances in the upper gastrointestinal tract usually affect the stomach more than the mouth, as food remains in the mouth for a shorter time than it remains in the stomach and subsequently enters the small intestine. The most important property of prebiotics in the upper gastrointestinal tract is that they survive the stomach due to its highly acidic environment. An important characteristic of prebiotics is that the nutrients are not destroyed in the stomach. When these nutrients are destroyed in the upper digestive tract, they do not reach the lower gastrointestinal tract, which contains many beneficial microorganisms that can use these undigested nutrients for growth.

When comparing the protective effect of AOSs on the three LAB strains under the same conditions, it was found that AOSs best protected *L. plantarum* under α-amylase conditions and *L. acidophilus* under HCl conditions. All LAB strains exhibited the lowest survival rate under bile salt conditions. Moreover, AOSs maintained the survival rate of *L. plantarum* at 1.85% after 60 min of incubation under bile salt conditions, which was five times higher than that of GOSs. Therefore, in further studies, it would be interesting to investigate how to improve resistance under bile salt conditions, e.g., using encapsulation techniques to increase resistance under simulated gastrointestinal conditions

## 4. Materials and Methods

### 4.1. Microorganisms

Three strains of LAB, *Lactobacillus acidophilus* TISTR 1338, *L. casei* TISTR 1463, and *L. plantarum* TISTR 1465, were obtained from the Thailand Institute of Scientific and Technological Research (TISTR), Thailand. The pathogenic bacteria, *Bacillus cereus* ATCC 11,778 and *Escherichia coli* ATCC 25,922, were obtained from the American Type Culture Collection, Manassas, VA, USA. All microbials used in this study were maintained at the Prebiotic and Probiotic Laboratory, Faculty of Science and Technology, Thammasat University.

### 4.2. Seaweed

Dry samples of *Gracilaria fisheri* used in this study were acquired from a seaweed farm in Songkhla Province, Southern Thailand, in November 2021. The sample was soaked in water for 3–5 h, washed several times with tap water to remove impurities such as sediments, epiphytes, and salts, and then dried in the shade. The dried sample was cut into small pieces (0.5–1 cm), packed in Ziploc plastic bags, and stored at room temperature.

### 4.3. Agar Polysaccharide (APS) Extraction

The dried seaweed (30 g) was extracted in an autoclave with 1 L of distilled water and heated at 110 °C for 90 min. The extract was then filtered through muslin cloth to separate the algae residues and the extract. The APS extract was filtered using Whatman filter paper No. 2 (GE Healthcare Inc., Chicago, IL, USA). The extract was evaporated at 50 °C until the volume was 1/4 of the initial volume using a rotary evaporator. The APSs were dialyzed (molecular weight cut off: 12–14 kDa) with distilled water for 24 h to remove the salt and small molecules, following which they were freeze-dried, and the APS powder was packed in vacuum bags and stored at 4 °C.

### 4.4. Agar Oligosaccharide (AOS) Production

The agar oligosaccharide production process via enzymatic hydrolysis was carried out following the method by Putri et al. [15] with some modifications. The APS powder was dissolved in 50 mM potassium phosphate buffer (pH 6.0) to prepare APS solutions (0.5 g/mL, 1.0 g/mL, 1.5 g/mL, and 2.0 g/mL). The APSs were incubated with 10 units/mL β-agarase (EC number 3.2.1.81, Sigma-Aldrich, Waltham, MA, USA) at 45 °C for 0 min, 30 min, 60 min, and 120 min, and then centrifuged (MPW-380R, Lilienthal, Germany) at 4 °C for 15 min to collect AOSs.

### 4.5. Chemical Constituent Analysis

The total sugar content was analyzed according to Dubois et al. [38], using galactose as a standard. The reducing sugars were analyzed using the 3,5-dynitrosalicylic acid (DNS) method [39]. The oligosaccharide content was calculated by subtracting the content of reducing sugars from the total sugar content [40]. The sample (20 mg) was hydrolyzed with 2 N HCl at 100 °C for 2 h, and then the sulfate content was measured according to Craigie et al. [41], using K_2_SO_4_ as a standard. The uronic acid content was determined according to Cesaretti et al. [42], using D-glucuronic acid as a standard. The protein content of polysaccharides was measured according to the Bradford method [43], using bovine serum albumin (BSA) as a standard.

### 4.6. Monosaccharide Composition Analysis

The monosaccharide compositions of APSs and AOSs was determined via HPAEC-PAD (Dionex™ ICS 5000, Thermo Scientific, Carlsbad, CA, USA). The APS and AOS samples were hydrolyzed according to Wahlström et al. [44]. The samples were hydrolyzed with 2 M methanolic HCl at 100 °C for 5 h. The solution was then neutralized with pyridine. The solvent in the reaction tube was evaporated by blowing with N_2_ gas. The dried sample was hydrolyzed with 2 M trifluoroacetic acid (TFA) at 120 °C for 1 h. The monosaccharides were analyzed on a CarboPac PA20 column (4 × 250 mm) with a guard column (50 mm × 4 mm) at a flow rate of 0.5 mL/min with 0.3 M NaOH. A linear gradient was applied for 20 min by adding deionized water and for a further 16 min by mixing solutions of 0.2 M NaOH and 0.2 M NaOH with 0.17 M sodium acetate at a flow rate of 1.0 mL/min. A mixture of arabinose, rhamnose, galactose, glucose, xylose, and mannose was used as a standard. A calibration curve was generated for each analyte and quantified using a linear regression equation. The data were processed and analyzed using Chromeleon^®^ software (v.6.8).

### 4.7. Molecular Weight Distribution Analysis

The molecular mass distributions of APSs and AOSs were analyzed via gel permeation chromatography (GPC) (LC-20A series, Shimadzu, Japan), which was specifically used to analyze the molecular weights of APSs and AOSs according to the method of Hung et al. [12] with some modifications. The HPLC system was equipped with Shodex Ohpak SB-806M HQ columns (8 mm × 300 mm; particle size 13 µm) at 30 ± 1 °C and a refractive index (RI) detector. Elution was performed with 0.02% NaN_3_ at a flow rate of 0.25 mL/min. The 10 µL sample (5 mg/mL) was injected for 70 min. The standard curve was generated using the pullulan standard, with a molecular weight of 180–805,000 Da (ReadyCal-Kit Pullulan high).

### 4.8. FT-IR Analysis

The functional groups of APSs and AOSs were investigated using Fourier-transform infrared (FT-IR) spectroscopy equipped with an attenuated total reflectance crystal accessory (ATR Golden Gate) (PerkinElmer Spectrum IR 10.6.2., Waltham, MA, USA) in transmission mode at wavenumbers ranging from 4000 cm^−1^ to 400 cm^−1^.

### 4.9. Prebiotic Properties Determination

#### 4.9.1. LAB Preparation

To investigate the prebiotic properties of AOSs, LAB (*L. acidophilus*, *L. casei*, and *L. plantarum*) were anaerobically incubated at 37 °C for 48 h in different culture compositions. The De Man Rogosa and Sharpe (MRS) broth with 50 mM citrate/phosphate buffer (pH 6.0) and mineral salt broth was used as a control group. Galacto-oligosaccharides (GOSs, Sigma-Aldrich), a commercially available probiotic, were added to the MRS broth and was used as a positive control group; MRS broth containing 0.5%, 1.0%, and 1.5% (*v*/*v*) of AOSs (0.3 mg/mL) was used as the test group.

#### 4.9.2. Prebiotic Effect on LAB Growth

The LAB were incubated at 37 °C for 6 h to stimulate growth. The diluted solutions obtained via serial dilution were cultured in triplicate on MRS agar to count the LAB growth colonies [44]. The growth-enhancing activity was calculated using Equation (1):

(1) $$\mathrm{Enhancing}\;\mathrm{activity}\;(\%)=\frac{\left[A-B\right]}{B}\times 100\%$$

where A is a LAB colony of a test group (log CFU/mL), and B is a LAB colony of a control group (log CFU/mL).

#### 4.9.3. Inhibitory Effect on Pathogenic Bacteria

The inhibitory effect of AOSs was determined according to the method of Wongsiridetchai et al. [45] with some modifications. The pathogenic bacteria (*B. cereus* and *E. coli*) were cultured at 37 °C for 24 h to prepare the inoculum. The inoculum (1% *w*/*v*) was transferred individually to the nutrient broth (NB), with mineral sal broth and 50 mM citrate/phosphate buffer (pH 6.0) used as a control group. NB with 0.3 mg/mL GOSs served as the positive control group, and NB with 0.5%, 1.0%, and 1.5% (*v*/*v*) AOSs (0.3 mg/mL) was used as the test group. The cultures were then incubated at 37 °C for 6 h. The diluted solutions obtained via serial dilution were cultured on nutrient agar (NA) using the spread plate technique. The ability of AOSs to inhibit pathogens was determined by measuring the absorbance at 600 nm and performing calculations using Equation (2):

(2) $$\mathrm{Inhibitory}\;\mathrm{activity}\;(\%)=\frac{\left[B-A\right]}{B}\times 100\%$$

where A is the optical density at 600 nm (OD_600_) of the test group, and B is OD_600_ of the control group.

#### 4.9.4. Protective Effect of AOSs under Simulated Gastrointestinal Conditions

The simulated gastrointestinal condition was prepared using the method of Sawangwan [46] with some modifications. The LAB cells from the LAB preparation (4.9.1) were washed twice with phosphate buffer saline (pH 7.0) and then centrifuged for 15 min at 8000× *g* rpm at 4 °C. The cell pellets were incubated with 100 units/mL α-amylase at 37 °C for 0, 10, and 15 min as an oral condition. Incubation with 0.1 M hydrochloric acid (HCl) at 37 °C for 0, 30, 60, 120, and 180 min as a gastric condition. For the small intestine conditions, the cell pellets were incubated with 0.3% bile extract at 37 °C for 0, 30, 60, 120, and 180 min and with PBS (pH 7.0) as control. The spread plate technique was used to determine the survival rate of LAB under simulated gastrointestinal conditions. The survival rate of LAB was calculated using Equation (3):

(3) $$\mathrm{Survival}\;\mathrm{rate}\;(\%)=\frac{Survival\;inoculum}{Initial\;inoculum}\times 100$$

### 4.10. Statistical Analysis

The data on chemical composition and prebiotic properties were expressed as mean values from triplicate determination ± standard deviation. Statistical differences between the mean values were determined via one-way ANOVA followed by Duncan’s multiple range test using SPSS Statistics v. 28 software. *p*-values less than 0.05 (*p* < 0.05) were considered statistically significant.

## 5. Conclusions

Seaweed oligosaccharides with prebiotic potential have recently attracted the attention of the nutraceutical and functional food industry. In this study, AOSs were obtained from the agar of *G. fishery* via hydrolysis with agarases. The physicochemical constituents of AOSs were determined in terms of total carbohydrates, reducing sugars, sulfate, uronic acid, protein, monosaccharides, and molecular mass. The chemical structure was analyzed via FT-IR spectroscopy. The results showed that AOSs exhibited remarkable prebiotic properties. The addition of AOSs had a positive effect on growth and showed a protective effect on LAB (*L. acidophilus*, *L. casei*, and *L. plantarum*) under simulated gastrointestinal conditions. In addition, AOSs exhibited pathogen inhibitory activity against two pathogens, *B. cereus* and *E. coli*. Our results suggest that the consumption of probiotic bacteria with AOSs can promote the growth of prebiotics, protect probiotics when they pass through the gastrointestinal tract, and may generate an antipathogenic effect on bacteria. However, the probiotic properties of these AOSs need to be studied in gastrointestinal tract models to confirm their efficacy. Purification of oligosaccharides also need to be studied further.

## Figures and Tables

**Table 1:** Monosaccharide constituents (% of total sugar) of APS and AOS.

	APS	AOS
Glucose	12.58 ± 1.33	12.52 ± 0.20
Galactose	77.69 ± 4.87	70.95 ± 1.15
Rhamnose	0.64 ± 0.04	0.52 ± 0.07
Mannose	0.59 ± 0.02	0.53 ± 0.01
Xylose	3.69 ± 0.23	3.12 ± 0.22
Arabinose	nd	nd

Data are presented as the mean of triplicate determination ± standard deviation. APS: agar polysaccharide; AOS: agar oligosaccharide; and nd: not detected.

**Table 2:** The molecular weight distribution of APSs and AOSs.

Number of Peaks	Peak Area (%)	Mn (Da)	Mw (Da)	PI (Mw/Mn)
Agar polysaccharides (APSs)				
1	100	1.355 × 10^4^	4.816 × 10^6^	355.48
Agar oligosacharides (AOSs)				
1	49.38	2.215 × 10^4^	2.715 × 10^4^	1.22
2	50.62	1346	1756	1.30

Data are presented as the mean of triplicate determination ± standard deviation. Mn: number-average molecular weight; Mw: weight-average molecular weight; and PI: polydispersity index.

## Data Availability

Data are available within the article.
